# *Ex vivo* signatures of myocardial edema by *in vivo* T2-CMR in a novel large animal model of NSTE-ACS

**DOI:** 10.1186/1532-429X-15-S1-P124

**Published:** 2013-01-30

**Authors:** Henry Chang, Tam Tran, George Billman, Mark W Julian, Robert Hamlin, Orlando P Simonetti, Giuseppe Ambrosio, Elliott Crouser, Subha V Raman

**Affiliations:** 1 Ohio State University, Columbus, OH, USA; 2 University of Perugia, Perugia, Italy

## Background

Non ST-segment acute coronary syndromes (NSTE-ACS) comprise 70% of all ACS. Difficulty in recognizing which patients have myocardium at risk contributes to heterogeneity in NSTE-ACS management, leading to poor outcomes compared to ST-elevation myocardial infarction (STEMI). To date, lack of suitable animal models has impeded progress in optimizing care. We developed a novel canine model to mimic NSTE-ACS, and evaluated ex vivo myocardial characteristics in the context of in vivo T2-CMR that may identify at-risk and salvageable regions.

## Methods

Five mongrel dogs underwent thoracotomy to create a left circumflex coronary artery stenosis without complete occlusion and to place epicardial pacing electrodes, and two dogs underwent sham thoracotomy. Pacing the animals with circumflex stenosis produced ST depression that resolved upon termination of pacing. Pre- and post-pacing CMR was performed in anesthetized, intubated animals on a 1.5 Tesla scanner with T2 mapping and late gadolinium enhancement (LGE) scans.

## Results

T2 increased significantly (Figure) in the circumflex-supplied lateral wall post-pacing in animals with stenosis compared to their remote myocardium (54.9 ± 6.3 vs. 43.4 ± 3.6 ms, p < 0.001) without producing evident LGE-positivity; no such T2 increase occurred in sham-operated animals. Ex vivo measurements of cellular respiration revealed decreased respiration in ischemic vs. remote regions (0.44 ± 0.13 vs. 0.60 ± 0.09 nmol O2/mg/min, p < 0.01). Electron microscopy showed mitochondrial swelling in the ischemic region, but no membrane breaks or other signs of cellular necrosis.

**Figure 1:**
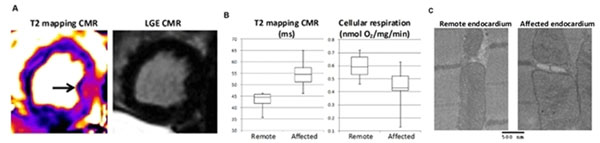
A) In a canine model of NSTE-ACS, T2 increased significantly in the lateral myocardium without evident LGE-positivity. B) Decreased cellular respiration paralleled increased T2, suggesting mitochondrial dysfunction. C) Electron microscopy of the endocardium showed normal mitochondria in the remote region and swollen mitochondria in the affected region without signs of cellular necrosis.

## Conclusions

These preliminary studies suggest that *in vivo* T2-CMR can identify regions of that heart with potentially reversible myocardial and mitochondrial injury in a novel canine model of NSTE-ACS.

## Funding

DHLRI, AHA

